# Novel copper complex CTB regulates methionine cycle induced TERT hypomethylation to promote HCC cells senescence via mitochondrial SLC25A26

**DOI:** 10.1038/s41419-020-03048-x

**Published:** 2020-10-11

**Authors:** Chun Jin, Yujia Li, Ying Su, Zijian Guo, Xiaoyong Wang, Shijun Wang, Feng Zhang, Zili Zhang, Jiangjuan Shao, Shizhong Zheng

**Affiliations:** 1 Jiangsu Key Laboratory for Pharmacology and Safety Evaluation of Chinese Materia Medica, Nanjing University of Chinese Medicine, Nanjing, 210023 China; 2 State Key Laboratory of Coordination Chemistry, School of Chemistry and Chemical Engineering, Nanjing University, Nanjing, 210023 China; 3 State Key Laboratory of Pharmaceutical Biotechnology, School of Life Sciences, Nanjing University, Nanjing, 210023 China; 4 Shandong Co-innovation Center of TCM Formula, College of Traditional Chinese Medicine, Shandong University of Traditional Chinese Medicine, Jinan, 250000 China; 5 Jiangsu Key Laboratory of Therapeutic Material of Chinese Medicine, Nanjing University of Chinese Medicine, Nanjing, 210023 China

**Keywords:** Cancer metabolism, Cancer metabolism

## Abstract

Related research has recognized the vital role of methionine cycle metabolism in cancers. However, the role and mechanism of methionine cycle metabolism in hepatocellular carcinoma are still unknown. In this study, we found that [Cu(ttpy-tpp)Br_2_]Br (Referred to as CTB) could induce hepatocellular carcinoma cells senescence, which is a new copper complex synthesized by our research group. Interestingly, CTB induces senescence by inhibiting the methionine cycle metabolism of HCC cells. Furthermore, the inhibitory effect of CTB on the methionine cycle depends on mitochondrial carrier protein SLC25A26, which was also required for CTB-induced HCC cells senescence. Importantly, we found that CTB-induced upregulation of SLC25A26 could cause abnormal methylation of TERT and inhibited TERT expression, which is considered to be an essential cause of cell senescence. The same results were also obtained in vivo, CTB inhibits the growth of subcutaneously implanted tumors in nude mice and promoted the expression of senescence markers in tumor tissues, and interference with SLC25A26 partially offset the antitumor effect of CTB.

## Introduction

Hepatocellular carcinoma (HCC) is one of the malignant tumors with high mortality, and it lacks effective treatment^1,2^. Despite continuous progress in improving early detection and developing new treatment strategies, the prognosis for patients with HCC remains unsatisfactory^3,4^. Therefore, exploring the molecular mechanism of HCC progress is crucial for finding new therapeutic targets^5,6^.

Infinite proliferation is a crucial characteristic of tumor cells^7^, and senescence becomes an effective way to inhibit malignant transformation by limiting the cell’s replication lifespan^8^. The main characteristics of cell senescence are the irreversible growth stagnation and the resulting cell senescence-related phenotype, which is mainly manifested by the increased activity of senescence-related β-galactosidase (SA-β-Gal), cell cycle stagnation and so on^9–12^. In some cases, cell senescence may be more considerable than apoptosis in terms of inhibiting tumor growth^13^. We often directly induce tumor cells apoptosis or other death methods, and these strategies or drugs strongly suppress the malignant proliferation of tumor cells. However, these methods often cause extremely serious toxic and side effects due to the lack of drugs that completely target tumor cells. Thus, it may be an important anti-tumor strategy that inducing tumor cells to senescent state, and then targeting these senescent cells.

Metabolic heterogeneity is also a feature of tumor cells, and tumor cells maintain their infinite proliferation characteristics by regulating their metabolism and nutrient acquisition^14^. Unlike normal cells that are powered by mitochondrial oxidative phosphorylation, tumor cells are more likely to convert glucose to lactic acid by glycolysis although the mitochondria of most tumor cells are not damaged, which is also the famous Warburg effect^15,16^. The traditional concept is that glucose is the only energy substrate for tumor cells, but tumor cells rely on multiple metabolic pathways for energy, and glucose is not the only energy source of tumor cells^17,18^. In recent years, various types of amino acid metabolism have gradually attracted the attention of researchers. The role of cysteine, glutamine, phenylalanine, tryptophan, and arginine in immune regulation has a greater impact on malignant tumors^19–21^. Recent studies show that tumor cells, especially tumor-initiating cells, have a high activity of methionine cycle metabolism, which could be a new direction in the field of metabolic reprogramming^22^. Methionine interacts with ATP to generate S-adenosylmethionine (SAM) under the action of methionine adenosine transferase. SAM removes adenosine to produce S-adenosyl homocysteine (SAH) after interacting with the methyltransferase, and the homocysteine is supplied to the methyl group by N5-methyltetrahydrofolate to generate methionine again. The above is a complete methionine cycle. The methionine cycle regulates cells biological function by providing methyl units for various molecules such as DNA, RNA, and lipids to affect its methylation level^23^. SAM synthesized directly from methionine is the main methyl donor molecule in cell methylation. Mitochondrial carrier SLC25A26 is responsible for transporting SAM produced by the methionine cycle to mitochondria, which is essential for maintaining mitochondrial homeostasis^24^. Aberrant expression of SLC25A26 can lead to various mitochondrial dysfunction such as abnormal translation of mitochondria-related proteins or impaired cell function by regulating with methyl metabolism^25^. These findings suggest that SLC25A26 may interact with the methionine cycle to influence cell fate. A subtle link between methionine and cancers have been noted that methionine may be a key molecule in the single-carbon metabolism that helps cancer cells grow. However, little is known about the role of methionine cycle metabolism in inducing of HCC cells senescence.

Recently, our team synthesized a new compound [Cu(ttpy-tpp)Br_2_]Br (Referred to as CTB) by introducing a tri-phenyl-phosphine (TPP) group into the copper complex [Cu(ttpy)Br_2_]^26^. CTB has DNA cleavage activity, good water solubility, and the ability to partially target mitochondria. Our previous studies have shown that CTB-induced Drp1-mediated phagocytosis in HCC cells, which leads to abnormal HK2 function and inhibited glycolysis^27^. CTB, as a candidate anti-tumor compound, has not fully been understood the mechanism of CTB’s anti-hepatocellular carcinoma effects. Herein, we will discuss whether CTB could induce HCC cells senescence by regulating methionine cycle metabolism to exert antitumor effects.

## Materials and methods

### Cell culture and transfection

Human HCC cell lines HepG2 and Huh-7 were obtained from the Cell Bank of Chinese Academy of Sciences (Shanghai, China). Both HepG2 and Huh-7 were cultured in Dulbecco’s modified Eagle’s medium DMEM (Gibco™, 11960044) containing 10% fetal bovine serum (Wisent Biotechnology Co., Ltd., Nanjing, China) and 1% antibiotics (penicillin and streptomycin). SLC25A26 siRNA was obtained from KeyGEN Biotechnology Co. Ltd. (Nanjing, China). SLC25A26 overexpression plasmid was constructed by Heyuan Biology Co. Ltd. (Shanghai, China). The siSLC25A26 or SLC25A26 plasmid were diluted with opti-MEM and incubated for 5 min at room temperature, and performed the same operation on the transfection reagent Lipo2000. Then mixing the diluted reagents together and incubating at room temperature for 20 min. Observation of cells carrying fluorescence under the microscope indicates successful transfection.

### Analysis of HCC cells senescence

HCC cells senescence was determined by the SA-β-Gal staining kit (Beyotime, C0602). The culture supernatant of HCC cells was aspirated and washed with PBS, then added 1 mL of β-galactosidase staining fixative and fixed for 15 min at room temperature. Then the cell fixative was aspirated and washed three times with PBS for 3 min. According to A:B:C:X-Gal = 1:1:93:5, the appropriate amount of dying working solution was configured. HCC cells were incubated at 37 °C overnight and observed under ordinary light microscope. The results were from triplicate experiments.

### RNA isolation and real-time PCR analyses

Trizol reagent (Sigma, Saint Louis, MO, USA) was used to extract total RNA. The A260/A280 values (1.8–2.0) were used to assess the purity of total RNA. Take an equal amount of total RNA from each sample and reverse transcribe it into cDNA. Real-time PCR was performed using the SYBR Green Master Mix (Yeasen,11202ES03) according to the specification. The fold change in the mRNA level of the target gene associated with the constant control GAPDH was calculated. All primers (Gen Script Co., Ltd., Nanjing, China) used in this experiment are listed in Table 1.

**Table 1:** Real-time PCR primer sequence.

Gene (Human)		Sequence
GAPDH	Forward	5′-GACATCAAGAAGGTGGTGAAGC-3′
	Reverse	5′-TGTCATTGAGAGCAATGCCAGC-3′
HMGA1	Forward	5′-CAACTCCAGGAAGGAAACCA-3′
	Reverse	5′-AGGACTCCTGCGAGATGC-3′
p16	Forward	5′-GGAGTTAATAGCACCTCCTCC-3′
	Reverse	5′-TTCAATCGGGGATGTCTGAGG-3′
p21	Forward	5′-GTCAGTTCCTTGTGGAGCCG-3′
	Reverse	5′-GAAGGTAGAGCTTGGGCAGG-3′
TERT	Forward	5′-CGGAAGAGTGTCTGGAGCAA-3′
	Reverse	5′-GGATGAAGCGGAGTCTGGA-3′
MAT2A	Forward	5′-TAGCCTTGGAGCAACAGTCA-3′
	Reverse	5′-CCATTACGGCGTAGTTCTGC-3′
SLC25A26	Forward	5′-AGATAAACCAAAATGAAATATCC-3′
	Reverse	5′-TCAGACCTCGTCAAGGCTACAAT-3′

### Western blot analyses

RIPA buffer containing protease inhibitors (1%) and phosphatase inhibitors (1%) was used to prepare total cellular protein extracts from HCC cells or tumors. Protein detection and quantification were performed as we previously described^28^. β-actin was used as a constant control, loading total protein in equal amounts. Representative blots were shown.

### Immunohistochemistry assay

Immunostaining was performed as described previously^28^. In brief, the tissues were routinely dewaxed, rinsed, and the sections were placed in Hydrogen Peroxide Block for 10–15 min to reduce non-specific background staining caused by endogenous peroxidase. Next, the slides were treated with primary antibody Ki67, HMGA1 or SLC25A26 overnight at 4 °C. All the sections were added primary antibody enhancer dropwise and incubated at room temperature for 20 min, then rinsed twice and incubated in secondary antibody for 30 min. Sections were treated with DAB for 3–15 min. Finally, we observed the staining under the microscope.

### Immunofluorescence

The immunofluorescence experiment was performed according to our previous instructions^28^. DAPI was used to stain nuclei. Three fields were randomly selected under the microscope to be photographed (ZEISS Axio vert. A1, Germany). Representative views were shown in the figure.

### Flow cytometry

The cell cycle distribution was determined by PI staining and flow cytometry analysis. All the operations follow the instructions of the cell cycle staining kit (Nanjing Keygen Biotech Co., Ltd). HCC cells were collected and adjusted to 1 × 10^6^/mL, and 1 mL of single-cell suspension were prepared to treat. HCC cells were fixed with 500 µL of cold ethanol after removing the supernatant. All the samples were washed with PBS before staining. Next, HCC cells were added with 500 μL of pre-prepared PI / RNase A staining working solution and incubated for 30–60 min in the dark. Flow cytometry (FACS Calibur; Becton, Dickinson and Company, Franklin Lakes, NJ, USA) recorded the red fluorescence at the excitation wavelength of 488 nm. All experiments were repeated three times independently.

### Methionine metabolite analysis by HPLC

The cell-solvent mixture was centrifuged and the supernatant was dried in a vacuum centrifuge and then dissolved in water for HPLC analysis. All of the methionine metabolite measurements were performed on Ultra high-performance liquid chromatograph (Thermo Corporation, USA, Ultimate 3000). The column is ACQUITY UPLC HSS T3 (100 mm × 2.1 mm × 1.8 μm); Mobile phase: A (50 mmol/L NaH_2_PO_4_ with 10 mmol/L sodium heptane sulfonate, pH = 4.38): B (methanol) = 80:20; Column temperature 30 °C, wavelength 260 nm, injection volume 2 μL, running time 10 min.

### Methylation-specific PCR and high throughput sequencing-bisulfite sequencing PCR

Cellular genomic DNA was extracted with TIANamp Genomic DNA Kit (Tiangen Biotech, Beijing, CO., LTDZYMO) and modified by the Methylation-Gold Kit (Tiangen Biotech, Beijing, CO., LTDZYMO). Twenty microliters of genomic DNA from each sample was used for bisulfite treatment and PCR amplification. The entire PCR amplification system includes 10 × PCR buffer, 2.5 mM dNTPs, 10 μM gene primer, genomic DNA, and 5 U/μL Taq HS. Performing electrophoresis under 1 × TAE, 2.0% agarose, 5 V/cm electrophoresis. Primer sequences are listed in Table 2.

**Table 2:** Methylation-specific PCR primer sequence.

Gene (Human)	Sequence
H-TERT(M)-F	5′-CGTGGTTTTTCGTTTAGGACGT-3′
H-TERT(M)-R	5′-CGCGTATCCATCAAAACGTAAA-3′
H-TERT(U)-F	5′-ATATGTGGTTTTTTGTTTAGGATGTTG-3′
H-TERT(U)-R	5′-AAACCACATATCCATCAAAACATAAA-3′

DNA integrity and degradation degree were analyzed by agar-gel electrophoresis to confirm no significant degradation. Nanodrop was used to measure DNA purity: the OD260/280 ratio ranged from 1.8 to 2.0. The non-methylated C becomes U (T after PCR amplification), while the methylated C remains unchanged after Bisulfite (EZ DNA Methylation Gold Kit, Zymo Research) treatment. BSP amplification was performed on Bisulfite treated templates by high fidelity U-base resistant DNA polymerase. The BSP amplification products were mixed and amplified with label primers and illumina sequencing connector. Finally, each sample library was purified, quantified, mixed with various libraries, checked and sequenced by computer. The sequencing platform is PE150 and the sequencing volume is not less than 200X. Primer sequences are listed in Table 3.

**Table 3:** Bisulfite sequencing PCR primer sequence.

Gene (Human)	Sequence
BS6_D-loop (H)-F	5′-CACATCTCTACCAAACCCC-3′
BS6_D-loop (H)-R	5′-TGGGGTGATGTGAGTTTGTT-3′
BS6_D-loop (L)-F	5′-AGAGAGTATATTTTTGTTAAATTTT-3′
BS6_D-loop (L)-R	5′-AGGAAGAGAGACCCATCTAAACATTTTCAA-3′
mtCOX2 (L)-F	5′-ATTGGTTATTAATGGTATTGAATTTA-3′
mtCOX2 (L)-R	5′-CTCCACAAATTTCAAAACATTAAC-3′

### Measurement of tissue SAM levels

SAM levels in tissues were measured by kits (mlbio, ml038511) following the manufacturer’s instructions. To put it simply, set a standard curve, then the samples of different groups were added to the sample wells of the enzyme-coated plate. After incubation, washing, enzyme addition and color development, the absorbance of the sample was used to calculate the SAM relative level.

### Measurement of ATP levels

ATP levels in cells were measured by kits (Beyotime, S0026) following the manufacturer’s instructions. The cells were lysed by lysis buffer and the cell supernatant was extracted. ATP test solution was diluted in a proportional way and evenly added into the black 96-well plate. Samples and standards were quickly moved into the test hole and carefully mixed. Finally, the RLU value or CPM was measured at least 2 s later by luminometer or liquid flash.

### Measurement of liver ALT, AST levels

Liver levels of alanine aminotransferase (ALT), aspartate aminotransferase (AST) were measured using kits (Nanjing Jinting Bioengineering Institute, Nanjing, China) according to the protocols provided by the manufacture.

### Animals and experimental design

All experimental procedures have been approved by the Animal Protection and Use Agency of Nanjing University of Traditional Chinese Medicine (Nanjing, China), and all animals have been subject to humane care under the guidance of the National Institutes of Health (USA). Four weeks old male nude mice (BALB/C-nu/nu) purchased from the Nanjing Institute of Biomedicine (Nanjing, China). All mice were randomly distributed in cages (5/cage) for 5 days of adaptive feeding. Huh-7 cells with logarithmic growth (cell density 2 × 10^7^ cells/200 µL) were inoculated on the right side of mouse to construct a subcutaneous xenograft model. The long diameter (*a*) and short diameter (*b*) of were measured with vernier caliper. The tumor volume was expressed as (*V*) = *a* × *b* ^2^/2. All the mice were divided into five groups (six animals per group). Group 1 was treated as the model group. Group 2 was treated as the blank interference group. Both groups 3 and 5 were treated as CTB (5 mg/kg) intraperitoneal injection groups. The mice in group 4,5 were interfering with SLC25A26.

### Reagents and antibodies

CTB was provided by the State Key Laboratory of Coordination Chemistry at Nanjing University^26^. Compound SAMe (S-(5′-Adenosyl)-L-methionine tosylate) (T4447) was purchased from Target Mol (Shanghai, China). Compound 5-Aza (5-Aza-2′-deoxycytidine) (T1508) was purchased from Target Mol (Shanghai, China). Dulbecco’s modified minimal medium (DMEM), fetal bovine serum (FBS) and Opti-MEM medium were purchased from GIBCO BRL. All antibodies were used after dilution according to the manufacturer’s instructions. Antibodies: p16 (A0262), p21 (A1483), TERT (A16625), Ki67 (A11005) and β-actin (AC026) were purchased from Abclonal (Wuhan, China); HMGA1 (382971) were obtained from ZEN BIO (Chengdu, Sichuan); CDK4 (11026-1-AP), CDK6 (14052-1-AP), cyclinD1 (60186-1-Ig), cyclinE1 (11554-1-AP) were obtained from Proteintech Group (Wuhan, China); MAT2A (PA5-72927) were obtained from Invitrogen (California, America); SLC25A26 (PA5-55452) and SLC25A26 (ab175209) were purchased from Invitrogen and Abcam (Shanghai, China) respectively. Horseradish peroxidase-conjugated anti-mouse and rabbit secondary antibodies were obtained from Proteintech Group (Wuhan, China).

### Statistical analysis

Statistical analysis was determined by the one-way analysis of variance by Student’s *t* test (comparison of two groups) or Student-Newman-Coors test (more than two groups). All data were analyzed with GraphPad Prism 8.0. Data were indicated as means ± S.D. Differences were considered as significant (*P < 0.05); very significant (**P < 0.01) and highly significant (***P < 0.001).

## Results

### CTB promoted HCC cells senescence in vitro

**Fig. 1:**
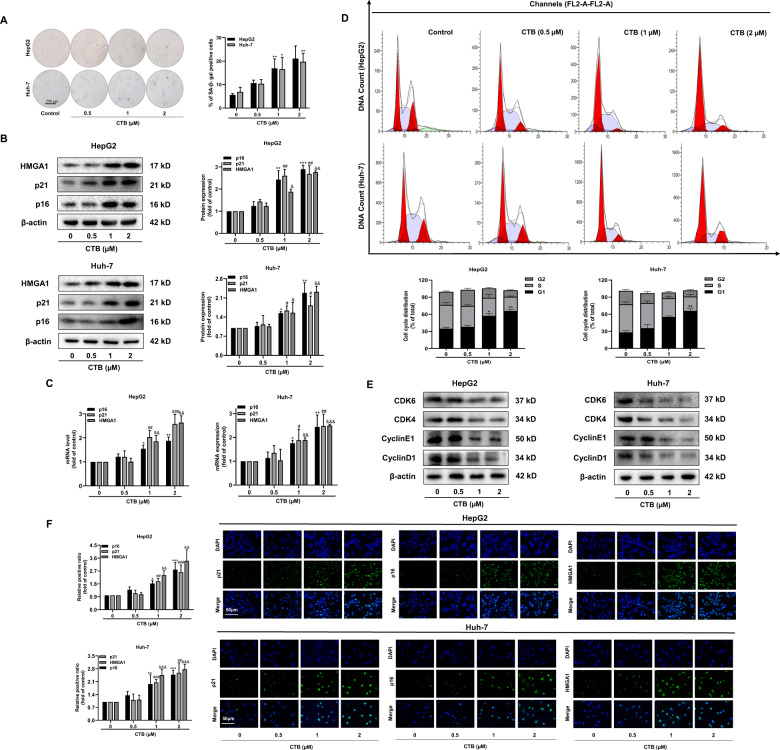
CTB promoted HCC cells senescence in vitro. HepG2 cells and Huh-7 cells were incubated with the prescribed concentration of CTB for 24 h. **A** The senescence-related β-galactosidase staining kit was used to detect the proportion of senescent cells. Scale bars are 200 μm; **B**, **C** Western blot and real-time PCR were used to quantify the protein and mRNA levels of senescent markers p16, p21, and HMGA1. Graphic imprinting results were derived from three separate experiments. Statistical significance for this graph, data are represented as mean ± S.D. (*n* = 3); *P < 0.05 vs. control (p16), **P < 0.01 vs. control (p16), ##*P* < 0.01 vs. control, ###*P* < 0.001 vs. control (p21), & *P* < 0.05 vs. control and && *P* < 0.01 vs. control (HMGA1); **D** Flow Cytometry analyzed cell cycle to determine the percentage of cell cycle distribution; **E** The expression of cell cycle-regulatory proteins CDK6, CDK4, CyclinD1, and CyclinE1 was detected by western blot; **F** Immunofluorescence in situ analysis of the expression of p16, p21, and HMGA1. The nucleus was stained by DAPI. Scale bars are 50 μm.

We cultured two liver cancer cell lines simultaneously to explore whether CTB could induce HCC cells senescence in vitro. The classic feature of cell senescence is the upregulation of senescence-associated β-galactosidase (SA-β-Gal) activity^9^. Our experimental results indicated that CTB treatment concentration-dependently upregulated the number of senescent cells. (Fig. 1A). Meanwhile, we detected the protein and mRNA levels of senescence-related makers p16, p21, and HMGA1 via western blot and real-time PCR. Correspondingly, the results suggested that CTB upregulated the expression of these molecules at both protein and mRNA levels (Fig. 1B, C). The same results were obtained from the immunofluorescence experiment (Fig. 1F).

Irreversible cell cycle arrest is another major feature of cell senescence in addition to the above indicators^9^. We analyzed the impact of CTB on the cell cycle distribution of HCC cells via using flow cytometry and the results showed that CTB increased the G1 phase ratio of HCC cells while decreasing the S phase ratio (Fig. 1D). We detected the expression of cyclin D1, cyclin E1, cyclin kinase CDK4, and CDK6 to further confirm the effect of CTB on the cell cycle of HCC cells. The results of western blot suggested that CTB concentration-dependently reduced the expression of these proteins (Fig. 1E). To sum up, these data indicated that CTB could promoted HCC cells senescence in vitro.

### CTB induces HCC cells senescence by inhibiting methionine cycle metabolism

**Fig. 2:**
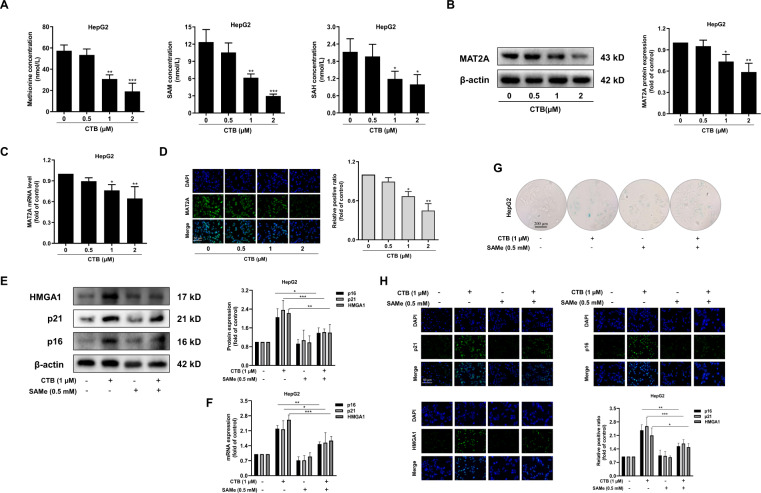
CTB-induced HCC cells senescence by inhibiting methionine cycle metabolism. HepG2 cells were incubated with the prescribed concentration of CTB or SAMe for 24 h. **A** Measurements of methionine circulating metabolites via HPLC; **B** The protein expression of MAT2A was detected by western blot; **C** Real-time PCR was used to quantify the mRNA level of MAT2A; **D** Immunofluorescence analysis of MAT2A. Scale bars are 50 μm; **E**, **F** Western blot and real-time PCR were used to quantify the protein and mRNA levels of senescent markers p16, p21, and HMGA1. Graphic imprinting results were derived from three separate experiments; **G** β-galactosidase staining kits were used to determine the relative content of senescent cells. Scale bars are 200 μm; **H** Immunofluorescence in situ analysis of the expression of p16, p21, and HMGA1. The nucleus was stained by DAPI. Scale bars are 50 μm. Statistical significance for this graph, data are represented as mean ± S.D. (*n* = 3); *P < 0.05 vs. control, **P < 0.01 vs. control, and ***P < 0.001 vs. control.

It is reported that cancer cells proliferation is highly dependent on the methionine cycle^29^. The high methionine cycle activity of cancer cells causes methionine to decompose beyond its synthetic ability, consequently causing tumor cells to become addictive to exogenous methionine^22^. We questioned whether CTB could influence methionine cycle. Next, we established a method for detecting methionine cycle metabolites SAM and SAH by HPLC. We observed that CTB treatment decreased methionine, SAM, SAH in HCC cells (Fig. 2A). We further examined the effect of CTB on the rate-limiting enzyme MAT2A of methionine cycle metabolism. The results suggested that CTB downregulated the expression of MAT2A in HCC cells at the protein and mRNA levels (Fig. 2B, C). The same result was obtained with immunofluorescence (Fig. 2D). These findings collectively revealed that CTB-inhibited methionine cycle activity in HCC cells.

We next asked whether inhibition of methionine cycle was associated with the HCC cells senescence induced by CTB. The methionine cycle metabolic supplement SAMe was used to test the pertinence. Western blot and real-time PCR analysis of senescent-related indicators showed that SAMe at 0.5 mM could reverse the effect of CTB (Fig. 2E, F). Similarly, SAMe treatment attenuated CTB-induced senescence-associated β-galactosidase activity in HCC cells (Fig. 2G). Examination of senescence-associated genes using immunofluorescence staining consistently exhibited that SAMe significantly downregulated the p16, p21, HMGA1 in HCC cells induced by CTB (Fig. 2H). In summary, the above data indicated that inhibition of methionine cycle by CTB resulted in the HCC cells senescence.

### CTB promotes SLC25A26 accumulation and induces mitochondrial dysfunction

**Fig. 3:**
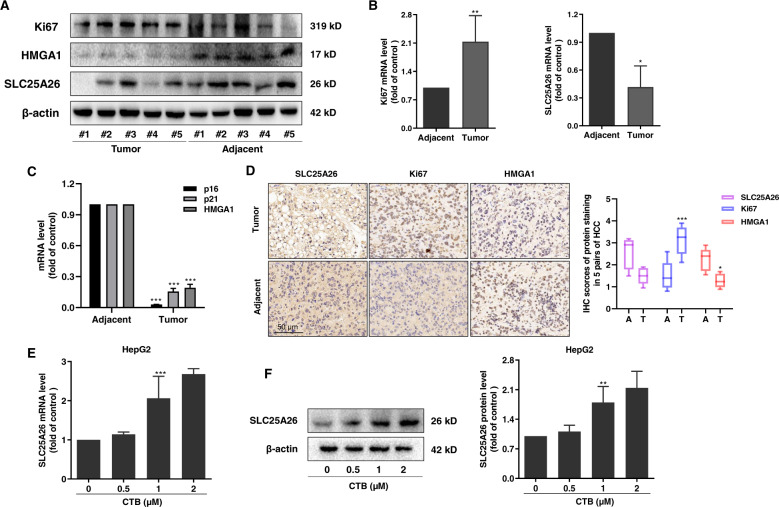
CTB promoted accumulation of SLC25A26. **A** Western blots were used to detect the protein expressions of Ki67, HMGA1, and SLC25A26 in liver cancer tissues and adjacent tissues from five pairs of HCC patients (T: tumor; A: Adjacent); **B**, **C** Real-time PCR analyses of Ki67, SLC25A26 and senescent makers in HCC tumor and adjacent tissues; **D** Typical images of Ki67, HMGA1 and SLC25A26 immunohistochemical staining in HCC tumors and adjacent tissues (scale bar, 50 μm); **E** The mRNA level of SLC25A26 was detected by real-time PCR. **F** Western blot analysis was used to determine the expression of SLC25A26. Statistical significance for this graph, data are represented as mean ± S.D. (*n* = 5); *P < 0.05 vs. control, **P < 0.01 vs. control.

SLC25A26 is a mitochondrial transport protein located in the mitochondrial inner membrane. The function of SLC25A26 is mainly to transport SAM produced by methionine cycle metabolism in the cytoplasm into the mitochondria^25^. SAM is a critical intermediate in the methionine cycle. Therefore, abnormal expression of SLC25A26 will affect the activity of methionine cycle metabolism. Relevant literatures reported that SLC25A26 showed low expression in both cervical cancer cells CaSki and HeLa^25^. We verified the expression of SLC25A26 in human clinical liver cancer tissues and adjacent tissues. The experimental results of western blot, real-time PCR and immunohistochemistry all showed that SLC25A26 had low expression in liver cancer tissues compared to adjacent tissues (Fig. 3A–D). Interestingly, the tumor tissues with low expression of SLC25A26 had high expression of proliferation index Ki67, while the senescence markers p16, p21, HMGA1 had low expression, and the tissue of high expression of SLC25A26 showed the opposite results (Fig. 3B–D). These data manifested that there may be a regulatory relationship between SLC25A26 and cell senescence.

Next, we focused on the regulation of SLC25A26 by CTB in HCC cells. We were surprised to find that CTB upregulated the expression of SLC25A26 mRNA in HCC cells (Fig. 3E). Western blot analysis also proved that CTB could up-regulate the protein level of SLC25A26 in HCC cells (Fig. 3F). Our previous research has shown that CTB possesses a certain inhibitory effect on mitochondrial respiration of HCC cells, which is manifested as a decreased OCR value^27^. Therefore, we investigated the effect of CTB-induced accumulation of SLC25A26 on mitochondrial function of HCC cells. The results of ATP content showed that CTB downregulated the mitochondrial ATP generation capacity of HCC cells, and siSLC25A26 treatment could partially offset the inhibitory effect of CTB on ATP generation (Supplementary Fig. 1A). The staining of JC-1 also indicated the loss of mitochondrial membrane potential after CTB treatment (Supplementary Fig. 1B). In general, these results indicate that CTB promoted the expression of SLC25A26 and induced mitochondrial dysfunction in HCC cells.

### The accumulation of SLC25A26 is essential for CTB to induce HCC cells senescence and inhibit the methionine cycle

**Fig. 4:**
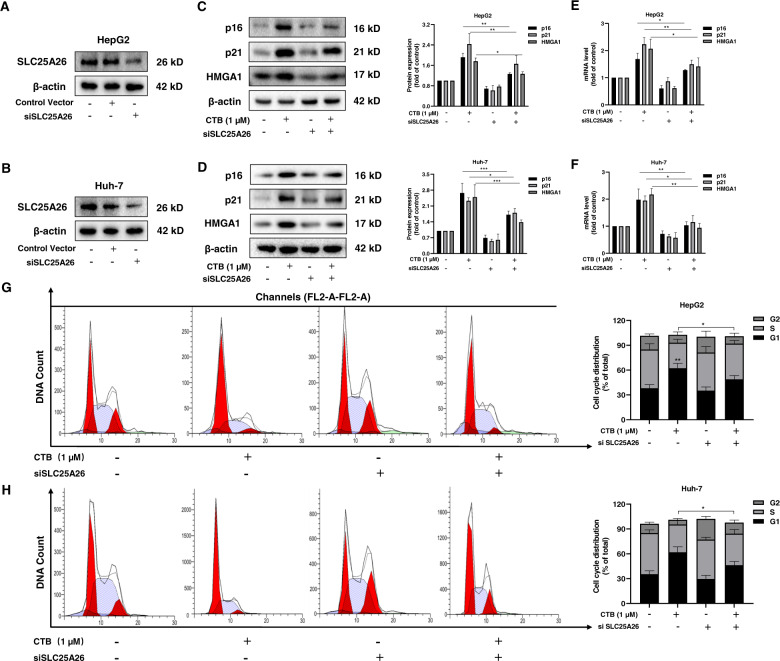
Interfering with SLC25A26 weakened the effect of CTB-induced HCC cells senescence. HepG2 cells and Huh-7 cells were incubated with the prescribed concentration of CTB or transfected with SLC25A26 siRNA for 24 h. **A**, **B** Western blot was used to analyze the transfection efficiency of siSLC25A26 in both HCC cells; **C**, **D** Western blot analyzed the expression of senescent makers; **E**, **F** Real-time PCR analyzed the expression of senescent makers; **G**, **H** Flow Cytometry analyzed cell cycle to determine the percentage of cell cycle distribution. Statistical significance for this graph, data are represented as mean ± S.D. (*n* = 3); *P < 0.05 vs. control, **P < 0.01 vs. control, ***P < 0.001 vs. control.

We speculate that SLC25A26 may play a key role in CTB-induced HCC cells senescence based on the results described in Fig. 3. To test this hypothesis, we pre-treated HCC cells with SLC25A26 siRNA or SLC25A26 plasmid, then added 1 µM CTB to culture HCC cells. We conducted subsequent experiments after verifying the transfection efficiency (Fig. 4A, B; Supplementary Fig. 2A, B). For the sake of exploring the impact of SLC25A26 on CTB-induced HCC cells senescence, the protein and mRNA levels of senescence markers p16, p21, and HMGA1 were examined. We found that overexpression SLC25A26 facilitated the expression of p16, p21 and HMGA1 both in protein and mRNA levels (Supplementary Fig. 2C–F), while SLC25A26 siRNA significantly inhibited the ability of CTB to induce HCC cells senescence (Fig. 4C–F). In addition, the results of flow cytometry also showed that CTB and SLC25A26 plasmids significantly increased the G1 phase ratio of HCC cells and reduced the S phase ratio (Supplementary Fig. 2G, H). Interestingly, pretreatment of HCC cells with SLC25A26 siRNA significantly counteracted the effect of CTB on cell cycle arrest in HCC cells (Fig. 4G, H). Overall, genetic deficiency of SLC25A26 effectively repress HCC cells senescence induced by CTB. On the contrary, overexpress SLC25A26 promotes HCC cells senescence and synergistic or antagonistic effects can be complied when used in combination with CTB. These results showed that the accumulation of SLC25A26 is essential for CTB-induced HCC cells senescence.

**Fig. 5:**
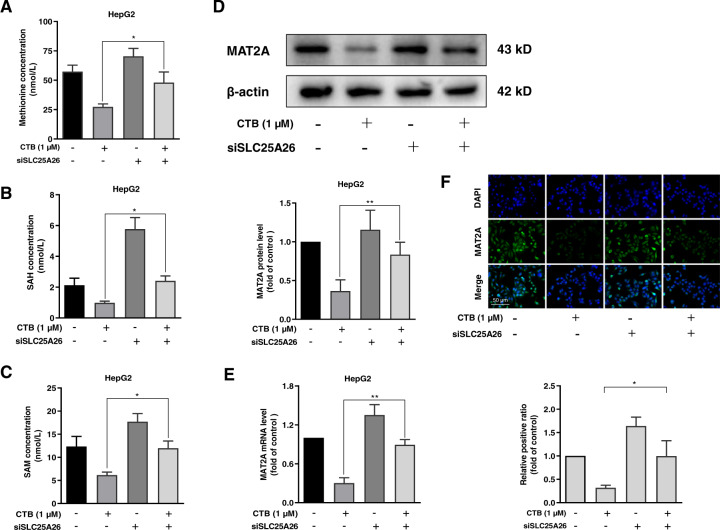
The accumulation of SLC25A26 was necessary for CTB to inhibit methionine cycle metabolism. HepG2 cells were incubated with the prescribed concentration of CTB or transfected with SLC25A26 siRNA for 24 h. **A**–**C** Measurements of methionine circulating metabolites via HPLC; **D** Western blot analysis of MAT2A protein expression with quantification; **E** Real-time PCR analysis of MAT2A mRNA level; **F** Immunofluorescence analysis of MAT2A. Scale bars are 50 μm. Statistical significance for this graph, data are represented as mean ± S.D. (*n* = 3); *P < 0.05 vs. control, **P < 0.01 vs. control.

We further investigated the association between the accumulation of SLC25A26 and inhibition of the methionine cycle by CTB in HCC cells. We applied the aforementioned method to measure the content of HCC cells methionine cycle metabolites SAM and SAH (Fig. 5A–C). The results showed that siSLC25A26 treatment canceled the inhibitory effect of CTB on HCC cells methionine cycle, which were confirmed after analyzing of methionine cycle metabolism rate-limiting enzyme MAT2A by western blot and real-time PCR (Fig. 5D, E). The same result was obtained by immunofluorescence (Fig. 5F). The above data indicate that the inhibition of CTB on the methionine cycle also requires the accumulation of SLC25A26.

### CTB-induced mitochondrial hypermethylation and TERT hypomethylation via mitochondrial SLC25A26

**Fig. 6:**
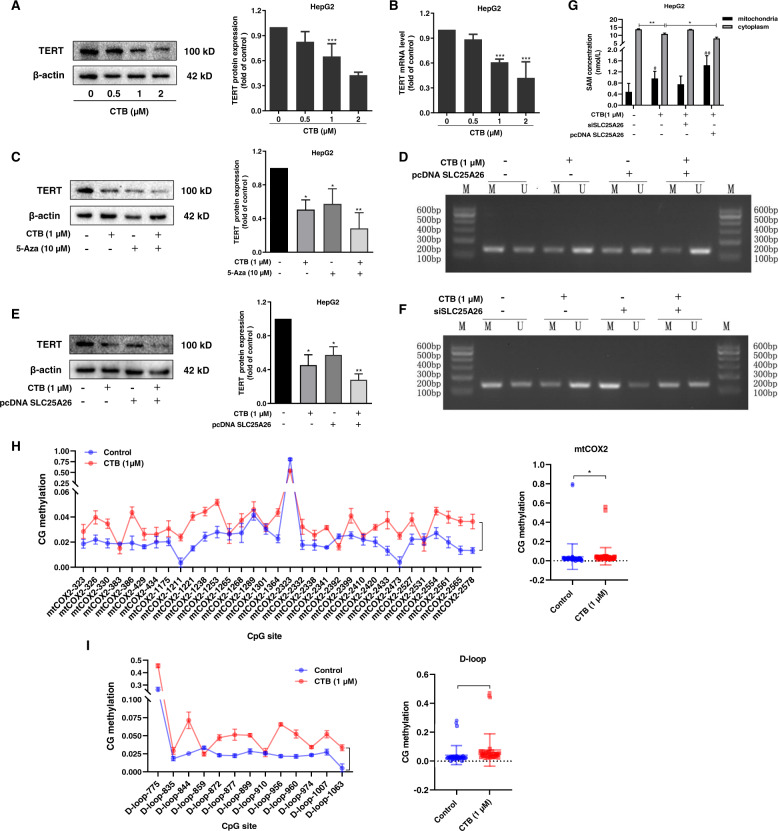
CTB-induced mitochondrial hypermethylation and TERT hypomethylation via mitochondrial SLC25A26. HepG2 were treated with CTB or 5-Aza at indicated concentrations and transfected with SLC25A26 plasmid or SLC25A26 siRNA for 24 h. **A**, **C**, **E** Western blot analysis of TERT protein expression with quantification; **B** Real-time PCR analysis of TERT mRNA level; **D**, **F** Methylation-specific PCR (MSP) analysis of TERT DNA methylation. **G** The SAM levels in HepG2 mitochondria and cytoplasm were detected by Elisa kit. **H** High-throughput BSP sequencing was performed to investigate the methylation levels of mitochondrial COX2 gene at various sites. **I** High-throughput BSP sequencing was performed to investigate the methylation levels of mitochondrial D-loop gene at various sites. Statistical significance for this graph, data are represented as mean ± S.D. (*n* = 3); *P < 0.05 vs. control, **P < 0.01 vs. control, ***P < 0.001 vs. control.

Based on the previous experimental results, we began to explore the possible mechanism of CTB regulating SLC25A26 to induce HCC senescence. Telomerase as a key factor that regulates senescence among variety mechanisms of senescence. Telomerase is similar to a cap at the end of a chromosome to protect the integrity of the information carrier DNA. Telomeres can prevent the loss of chromosomal DNA base pairs during continuous cell division. The length of the telomeres decreased after continuous division until the telomeres become too short to allow the cells to divide and give rise to cell senescence^30–32^. Therefore, we first measured the effect of CTB on the expression of telomerase reverse transcriptase (TERT) in HCC cells. Western blot and real-time PCR results showed that CTB could inhibit the expression of TERT, which were consistent with the previous experimental results (Fig. 6A, B).

Remarkably, another unique feature is that the TERT promoter is unmethylated in normal human cells and methylated in malignant cells. Cancer cells require TERT promoter methylation to activate TERT transcription, and TERT induction promotes abnormal methylation by up-regulating the expression of DNA methyltransferase, forming a positive feedback loop^33^. There is a transcription repressor binding site in the promoter region of TERT. There are some evidences that unmethylated TERT sites are beneficial for binding to transcription repressors to inhibit TERT expression^34^. Therefore, epigenetic changes are essential for the expression of TERT in tumors. To investigate whether the degree of TERT methylation is related to CTB inhibition of TERT-induced HCC senescence, we used DNA demethylating agent 5-aza-2-deoxycytosine (5-aza). Recent studies have found that demethylation with 5-aza-2-deoxycytosine could inhibit the expression of TERT gene and telomerase activity, and shorten the telomere length of cancer cells to play a certain anti-tumor effect^35^. We observed that 5-aza at 10 μM was similar to CTB at 1 μM, significantly inhibited the protein level of TERT, and combination of 5-aza and CTB produced a stronger reduction effect on TERT (Fig. 6C). CTB (1 μM) can effectively inhibited the level of TERT DNA methylation, which further confirmed our speculation (Fig. 6D, F). In addition, we also found that overexpression of SLC25A26 could produce a synergistic effect with CTB to inhibit the expression of TERT (Fig. 6E). Based on the previous experimental results, we speculate that CTB regulates of expression of SLC25A26 to inhibit the methionine cycle metabolism and affects the DNA methylation status of TERT. Then we conducted methylation-specific PCR (MSP) experiment to find that overexpression of SLC25A26 also inhibited the level of TERT DNA methylation (Fig. 6D). Interestingly, siSLC25A26 treatment partially canceled the inhibitory effect of CTB on TERT DNA methylation (Fig. 6F). Next, we asked the possible reasons for the effect of CTB on TERT methylation status. We hypothesized that the effect of CTB on SLC25A26 expression resulted in the imbalance of SAM levels in mitochondria and cytoplasm. We found that CTB promotes SAM accumulation in mitochondria and this effect could be partially offset by siSLC25A26 via measuring SAM concentration in mitochondria and cytoplasm (Fig. 6G). In addition, the results of BSP sequencing also showed that CTB treatment upregulated the methylation levels of D-loop (Fig. 6I) and mtCOX2 (Fig. 6H) spots of mitochondrial genes. In summary, these results indicate that CTB affects mitochondrial and cytoplasmic SAM levels by regulating SLC25A26 expression, thus affecting the methylation status of related genes.

### CTB repressed tumorigenesis in vivo by regulating SLC25A26

**Fig. 7:**
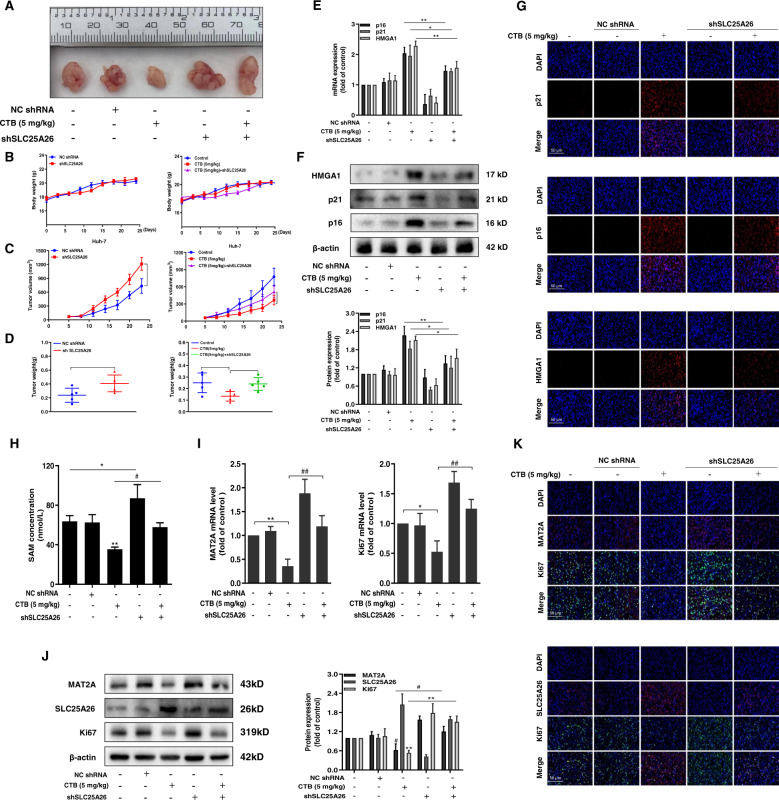
CTB repressed tumorigenesis in vivo by regulating SLC25A26. HCC cell line Huh-7 was used to construct subcutaneous xenografts. **A** Representative images of BALB/c nude mice tumors stripped from different groups (*n* = 6); **B** The body weight change curves of mice in each group during the whole experiment period (*n* = 6); **C** Tumor volume growth curves of mice in each group throughout the experimental period (*n* = 6); **D** Tumor weight was obtained after executing mice on the last day (*n* = 6); **E** Real-time PCR analysis of p16, p21, HMGA1 mRNA levels in tumor tissues; **F** Western blot was used to analyze the protein expression of p16, p21, HMGA1 in tumor tissues. Representative blots were from three independent experiments; **G** Immunofluorescence analyses of senescent makers p16, p21, HMGA1 in the tumor tissue; **H** Elisa kit analyzes SAM levels in the tumor tissue; **I** Real-time PCR analyses of MAT2A, Ki67 mRNA levels in tumor tissues. **J** Western blot was used to analyze the protein expression of MAT2A, SLC25A26, Ki67 in tumor tissues. Representative blots were from three independent experiments; **K** Immunofluorescence analyses of MAT2A, SLC25A26, Ki67 in tumor tissues; Scale bars are 50 μm. Statistical significance for this graph: *P < 0.05 vs. control, **P < 0.01 vs. control, #*P* < 0.05 vs. CTB (5 mg/kg) + shSLC25A26, ##P < 0.01 vs. CTB (5 mg/kg) + shSLC25A26.

We used the human hepatoma cell line Huh-7 to establish a subcutaneous xenograft model to verify the correlation in vivo. Because our recent research clearly shows that CTB has a powerful anti-hepatocellular carcinoma effect in vivo^27,36^. We focus on proving whether the role of CTB depends on regulating SLC25A26 in this section. The weight of all mice did not fluctuate greatly during the experimental period (Fig. 7B) and the indicators of liver damage ALT and AST also downregulated after CTB treatment (Supplementary Fig. 3), which suggested that CTB did not have obvious toxicity at this concentration. Next, we could see from the tumor growth curve that interfering with SLC25A26 significantly promoted tumor growth. The administration group remarkably inhibited the growth of tumors. Interestingly, the effects of CTB were abolished by interfering with SLC25A26 (Fig. 7A, C). At the same time, the same results were obtained by peeling and weighing the tumor weight of each mouse (Fig. 7D). Altogether, these observations indicated that CTB exerts anti-tumor effect by regulating SLC25A26 in mice.

We subsequently evaluated the effects of CTB on senescence in the xenograft model. We observed that senescence-related indicators in CTB-treated mice were significantly elevated compared to control mice, which was abrogated via interfering with SLC25A26 (Fig. 7E). Further detecting the protein levels of these indicators also received the same results (Fig. 7F). Immunofluorescence analyses revealed that these senescence markers and SLC25A26 had higher abundance in the HCC tissues of CTB-treated mouse compared to the model group, but interference with SLC25A26 impaired the effects of CTB (Fig. 7G). Next, we examined the effect of CTB on methionine cycle metabolism. Firstly, we detected the level of methionine circulating metabolite SAM. The results suggested that CTB administration group showed lower SAM levels, and the effect was canceled after interference with SLC25A26 (Fig. 7H). Subsequently, CTB administration reduced the protein and mRNA levels of MAT2A and Ki67, and all effects were canceled after interfering with SLC25A26 (Fig. 7I, J). Further immunofluorescence analysis also obtained the same results (Fig. 7K). Taken together, suppression of HCC methionine cycle metabolism by CTB contributed to the suppression of HCC in mice, and these effects were dependent on SLC25A26.

## Discussion

HCC is one of the most common causes of cancer-related deaths worldwide^4^. The onset of HCC is insidious and the early symptoms are atypical or difficult to diagnose. Only about 15% of patients are suitable for surgical resection, drug treatment is the main method. However, advanced patients often face the plight of no medicine, as a result, the prognosis is poor^37^. Cell senescence is a stress-responsive cell cycle arrest procedure, which could hinder the malignant expansion of cancer cells^8^. Therefore, pharmacological induction of tumor cell senescence has always been considered as one of the effective ways to hunt for anti-cancer drugs. Our recent research showed that CTB has a significant anti-hepatoma effect in vivo and in vitro. However, due to the limitations of chemotherapeutic drugs, we look forward to a more moderate way to achieve anti-tumor effects. We investigated whether low-dose CTB can induce HCC cells senescence. Cyclin-dependent kinase (CDK) inhibitors p16^INK4a^ and p21^Cip1^ are the key molecules of cell senescence. Our data suggested that CTB treatment upregulated the protein and mRNA levels of p16, p21, HMGA1 in HepG2 and Huh-7 (Fig. 1). Cell cycle distribution experiments also confirmed that CTB treatment could block liver cancer cells in G1 phase. These results indicated that CTB-inhibited HCC cells proliferation by inducing HCC cells senescence.

Metabolism plays an important role in maintaining intracellular homeostasis and responding to intracellular and extracellular stimuli by converting nutrients into metabolites^38^. As we all know, metabolic changes are one of the crucial characteristics of tumors. Tumor cells must adjust their metabolic methods in order to maintain continuous proliferation. The Warburg effect is a classic example of altering cancer metabolism. In addition to high glucose consumption, some tumors require high glutamine consumption to meet the metabolic needs of cancer cells^39^. A large number of basic and clinical trials have shown that targeting tumor-dependent amino acid metabolism and developing new drugs can effectively inhibit tumor growth^40^. Recently, Wai Leong Tam et al. identified abnormally active methionine cycle metabolism in tumor stem cells (TIS) through metabolomics analysis, and even short-term pharmacological intervention in methionine cycle metabolism can significantly inhibit the proliferation of tumor cells^22^. Therefore, the methionine circulating flux regulates the epigenetic state of tumor cells and drives tumorigenesis. Jason W Locasale et al. also confirmed that restricting the intake of methionine found in red meat and red eggs can significantly improve the cancer treatment effect of mice and slow down tumor growth^40^. Interestingly, low-dose chemotherapy itself has no effect on colorectal cancer, but it shows significant inhibition of tumor growth when combined with methionine restriction^41^. It has been recognized that methionine cycle metabolism was involved in many cellular functions, including methylation reactions, polyamine synthesis, and folic acid metabolism, coordination of nucleotide and redox states^42^. However, the specific role of methionine and one-carbon metabolism in tumor progression is unclear. At the same time, our results of HPLC show that CTB can significantly inhibit the activity of HCC cells methionine cycle metabolism (Fig. 2). The expression of MAT2A, which is the rate-limiting enzyme of methionine cycle metabolism, was also suppressed by CTB treatment. These results indicate that CTB downregulates the methionine cycle activity of HCC cells. Next, we examined the effect of methionine cycle on CTB-induced cell senescence. We used the methionine cycle supplement SAMe to enhance methionine cycle metabolism, and found that SAMe treatment abolished the effect of CTB upregulation of HCC cell senescence-related proteins (Fig. 2). The overall results indicate that CTB induce HCC cells senescence, while generally inhibiting the metabolism of methionine cycle in HCC cells. These findings drive us to explore the deeper mechanism of the interaction between cell senescence and methionine cycle metabolism.

We have established that CTB-induced cell senescence is caused by inhibiting the methionine cycle based on previous results. Next, our study identified the mitochondrial carrier family SLC25A26 as an upstream molecule that regulates CTB-induced HCC cells senescence. The physiological function of SLC25A26 is to transport the SAM produced by the methionine cycle to the mitochondria to maintain the mitochondrial methylation reaction. Menga et al.^25^ reported that SLC25A26 is overexpressed in CaSki cells. Highly expressed SLC25A26 promotes the level and utilization of mitochondrial SAM, induces high methylation of mtDNA, resulting in low expression of mitochondrial respiratory complex subunits and inhibits mitochondrial ATP generate. In addition, high levels of SAM in the mitochondria impair the cycle of the methionine cycle, greatly reducing the accumulation of homocysteine. In the present study, we found that overexpression of SLC25A26 promote CTB-induced HCC cells senescence, while further verification of the function of SLC25A26 suggested that interference with SLC25A26 partially canceled the effect of CTB-induced HCC cells senescence. Besides, the accumulation of SLC2526 was also essential for CTB-inhibited methionine cycle activity.

Given that the SAM produced by the methionine cycle is the only methylation donor for the whole-cell methylation reaction, we speculate that CTB inhibits methionine cycle metabolism and impairs methylation reaction to regulates HCC cells senescence. Many literatures reported that the activation of TERT was the main mechanism for tumor cells to escape aging and obtain malignant proliferation. DNA methylation of TERT promoter may be an important mechanism for TERT activation^43^. Ryo Nishikawa et al. research suggested that TERT promoter methylation may be a potential biomarker for predicting the recurrence of pituitary tumors^44^. We found that the DNA methylation inhibitor 5-aza could cooperate with CTB to inhibit TERT expression after confirming the inhibitory effect of CTB on TERT expression. The following MSP experiments further confirmed our hypothesis that CTB-induced HCC cells senescence by inhibiting TERT promoter methylation, and overexpression of SLC25A26 also inhibited TERT methylation levels.

**Fig. 8:**
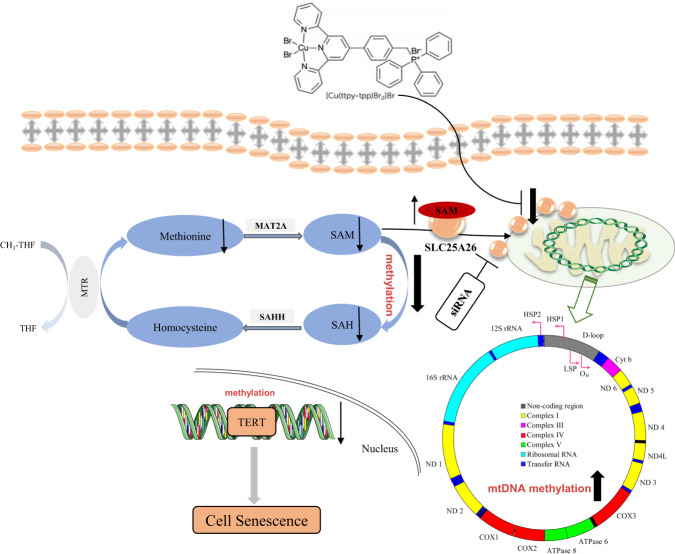
Mechanism diagram of CTB-induced HCC cells senescence. CTB, a novel copper complex, induced HCC cells senescence and promoted the expression of senescence-related indicators. Moreover, the effect of CTB on HCC cells senescence depends on its inhibitory effect on methionine circulation. Importantly, the mitochondrial transporter SLC25A26 plays a vital role in these effects. CTB affected the levels of methionine circulating metabolite SAM between cytoplasm and mitochondria by up-regulating the expression of SLC25A26 in HCC cells. These effects leaded to hypermethylation of mitochondrial genes and hypomethylation of TERT in cytoplasm. Ultimately, senescence of HCC cells occurred.

Finally, we verified the results of in vivo by constructing a subcutaneous xenograft model in nude mice. We obtained from the tumor growth curve that interfering with SLC25A26 significantly promoted tumor growth, which is consistent with our previous results. We detected senescence markers in tumors and found that interfering with SLC25A26 abolished CTB-induced cell senescence via western blot, real-time PCR, and immunofluorescence (Fig. 7). Overall, these results provide the first mechanistic evidence that the anti-HCC effect of CTB requires the interaction between methionine cycle metabolism and cell senescence (Fig. 8). However, this study also possessed certain limitations. We only preliminary discussed the effect of methionine cycle metabolism. The mechanism of methionine cycle metabolism on HCC cells senescence was relatively complex, but there is no doubt that targeting methionine cycle metabolism could play an anti-HCC effect.

## Supplementary information

Supplementary Figure Legends

Supplementary Fig. 1

Supplementary Fig. 2

Supplementary Fig. 3
