# Supplementary material for: Novel copper complex CTB regulates methionine cycle induced TERT hypomethylation to promote HCC cells senescence via mitochondrial SLC25A26
Source: Cell Death Dis. 2020 Oct 11;11(10):844. doi: 10.1038/s41419-020-03048-x (PMC7548283; doi:10.1038/s41419-020-03048-x)
Supplement: Supplementary file 1 — Supplementary Figure Legends [file 41419_2020_3048_MOESM1_ESM.docx]

**Supplementary Figure 1 The effects of CTB on mitochondrial biochemical indexes of HCC cells.**

HepG2 cells were incubated with the prescribed concentration of CTB and transfected with SLC25A26 siRNA or SLC25A26 plasmid for 24 h. (A). The production of mitochondrial ATP in HepG2 cells was detected by the kit. (B). The changes of mitochondrial membrane potential in HepG2 cells were detected by the JC-1 kit; Scale bars are 50 μm. Data are represented as mean ± S.D. (n=3); *P < 0.05 CTB vs. pcDNA SLC25A26, **P < 0.01 vs. control.

**Supplementary Figure 2** **Overexpression of SLC25A26 promoted CTB-induced HCC cells senescence.**

HepG2 cells and Huh-7 cells were incubated with the prescribed concentration of CTB or transfected with SLC25A26 plasmid for 24 h. (A and B). Western blot detected the transfection efficiency of SLC25A26 plasmid; (C and D). The protein content of senescent makers were detected by western blot; (E and F). The mRNA levels of senescent makers were detected by real-time PCR; (G and H). Flow Cytometry analyzed cell cycle to determine the percentage of cell cycle distribution. Statistical significance for this graph, data are represented as mean ± S.D. (n=3); *P<0.05 versus control, **P<0.01 versus control, ***P<0.001 versus control.

**Supplementary Figure 3 CTB downregulated the liver levels of hepatocyte injury markers (ALT, AST) in HCC mice.**

HCC cell line Huh-7 was used to construct subcutaneous xenografts. (A). The liver levels of ALT were measured by kits (n = 6); (B). The liver levels of AST were measured by kits (n = 6); *P<0.05 versus control, **P<0.01 versus control.
