# Supplementary figures and images for: Novel copper complex CTB regulates methionine cycle induced TERT hypomethylation to promote HCC cells senescence via mitochondrial SLC25A26
Source: Cell Death Dis. 2020 Oct 11;11(10):844. doi: 10.1038/s41419-020-03048-x (PMC7548283; doi:10.1038/s41419-020-03048-x)

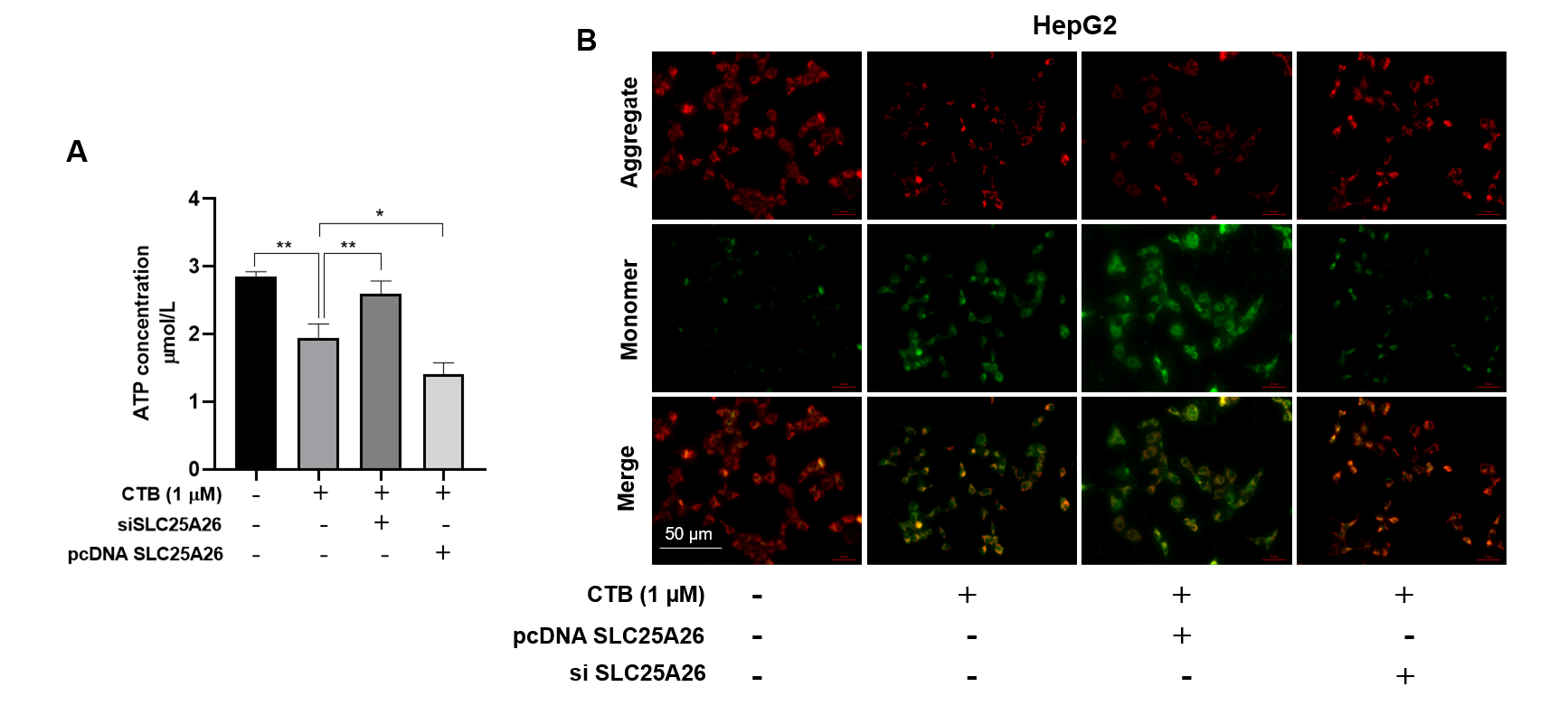

Supplement: Supplementary file 2 — Supplementary Fig. 1 [file 41419_2020_3048_MOESM2_ESM.tif]

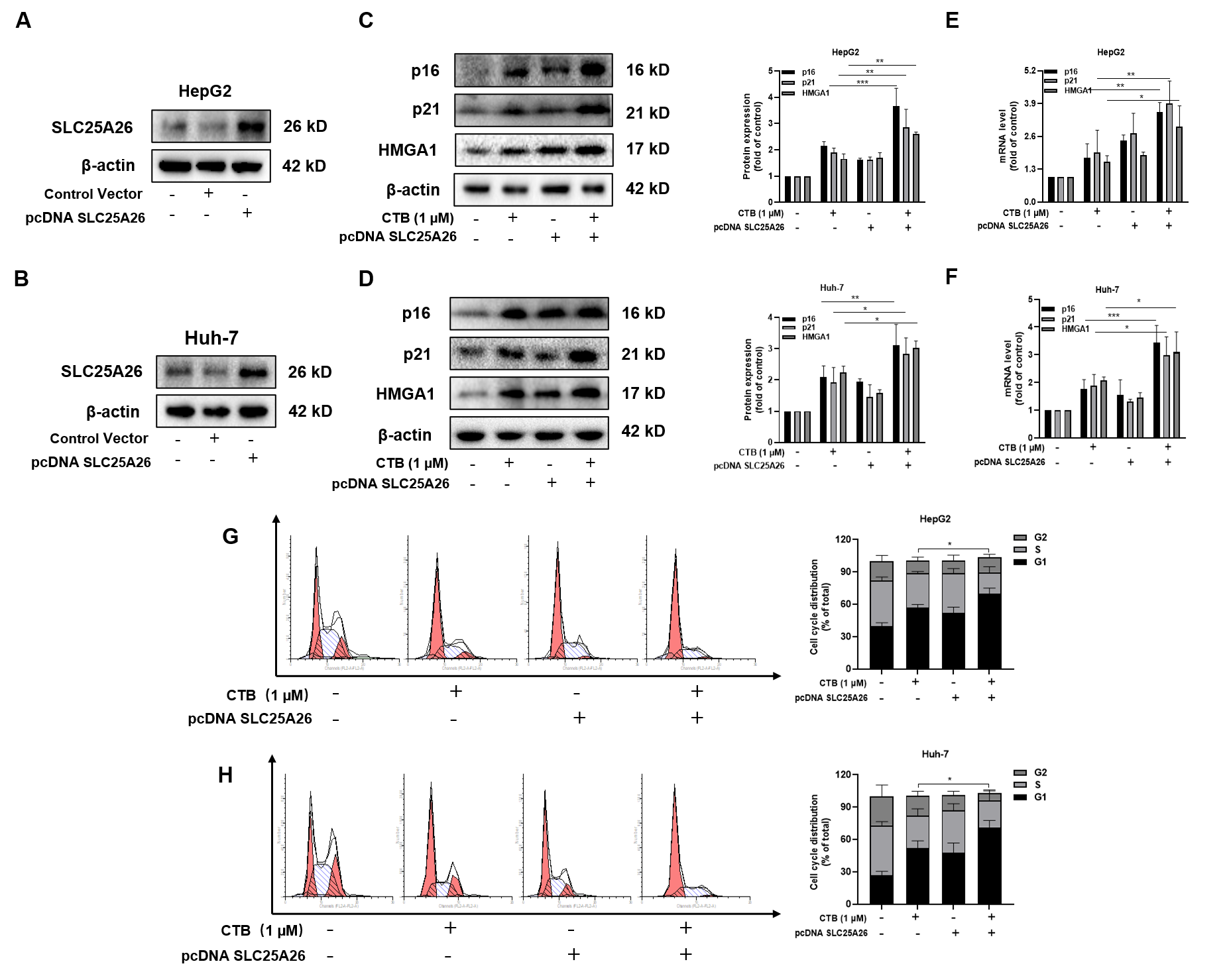

Supplement: Supplementary file 3 — Supplementary Fig. 2 [file 41419_2020_3048_MOESM3_ESM.tif]

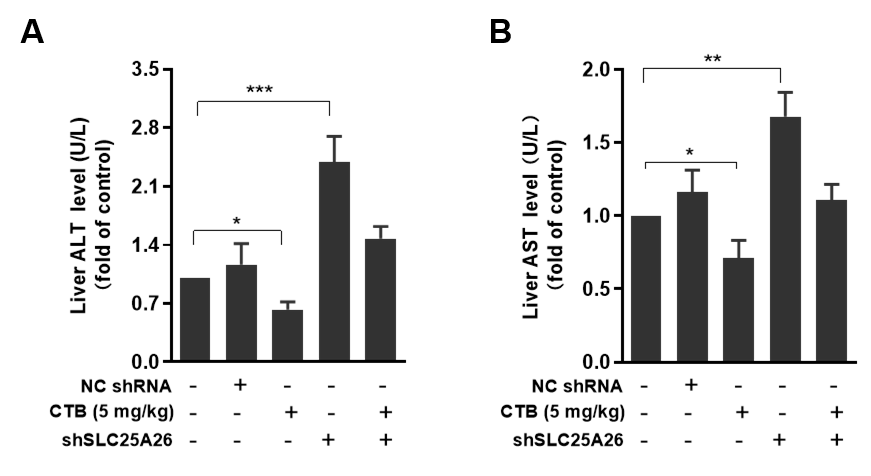

Supplement: Supplementary file 4 — Supplementary Fig. 3 [file 41419_2020_3048_MOESM4_ESM.tif]
